# Association between incidence of fatal intracerebral hemorrhagic stroke and fine particulate air pollution

**DOI:** 10.1186/s12199-019-0793-9

**Published:** 2019-06-01

**Authors:** Yifeng Qian, Huiting Yu, Binxin Cai, Bo Fang, Chunfang Wang

**Affiliations:** 1 Department of Oral and Craniomaxillofacial Surgery, Shanghai Ninth People’s Hospital, College of Stomatology, Shanghai JiaoTong University School of Medicine, Shanghai, China; 2 National Clinical Research Center for Oral Diseases, Shanghai, China; 3 Shanghai Key Laboratory of Stomatology and Shanghai Research Institute of Stomatology, Shanghai, China; 4 Department of Vital Statistics, Shanghai Municipal Center for Disease Control and Prevention, Shanghai, China; 5 Songjiang District Center for Disease Control and Prevention, Shanghai, China

**Keywords:** Fine particulate, Air pollution, Intracerebral hemorrhage, Effect modifiers

## Abstract

**Objective:**

Few studies investigating associations between fine particulate air pollution and hemorrhagic stroke have considered subtypes. Additionally, less is known about the modification of such association by factors measured at the individual level. We aimed to investigate the risk of fatal intracerebral hemorrhage (ICH) incidence in case of PM_2.5_ (particles ≤ 2.5 μm in aerodynamic diameter) exposure.

**Methods:**

Data on incidence of fatal ICH from 1 June 2012 to 31 May 2014 were extracted from the acute stroke mortality database in Shanghai Municipal Center for Disease Control and Prevention (SCDC). We used the time-stratified case-crossover approach to assess the association between daily concentrations of PM_2.5_ and fatal ICH incidence in Shanghai, China.

**Results:**

A total of 5286 fatal ICH cases occurred during our study period. The averaged concentration of PM_2.5_ was 77.45 μg/m^3^. The incidence of fatal ICH was significantly associated with PM_2.5_ concentration. Substantial differences were observed among subjects with diabetes compared with those without; following the increase of PM_2.5_ in lag2, the OR (95% CI) for subjects with diabetes was 1.26 (1.09–1.46) versus 1.05 (0.98–1.12) for those without. We did not find evidence of effect modification by hypertension and cigarette smoking.

**Conclusions:**

Fatal ICH incidence was associated with PM_2.5_ exposure. Our results also suggested that diabetes may increase the risk for ICH incidence in relation to PM_2.5_.

**Electronic supplementary material:**

The online version of this article (10.1186/s12199-019-0793-9) contains supplementary material, which is available to authorized users.

## Introduction

Stroke is listed as the second leading cause of death in the Global Burden of Disease, and the incidence of stroke is increasing, particularly in low- and middle-income countries, which accounts for two thirds of all strokes [1]. Evidence linking short-term exposure to outdoor air pollution with ischemic stroke (IS) became clearer, when the effect of air pollution on different types of stroke begun to be studied [2–8]. In recent years, several studies also reported associations between air pollutants and hemorrhagic stroke (HS), especially from studies conducted in Asia, where frequency of HS is substantially higher than that in Western countries [9–13].

Intracerebral hemorrhage (ICH) is a subtype of HS which results from rupture of small penetrating arteries deep in the brain [14]. Compared with the sizeable body of literatures concerning IS and HS, only a few of them have reported the association between air pollutants and ICH, and more importantly, limited studies have been conducted to examine the modification of such association by factors measured at the individual level, which presented an obstacle to a better understanding of the biological mechanisms of their association [14–19].

As the largest developing country, China is facing severe air pollution problem in recent years [9]. The objective of the present study was to investigate the influence of exposure to PM_2.5_ (particles ≤ 2.5 μm in aerodynamic diameter) on incidence of fatal ICH and to detect possible susceptible population in Shanghai, the largest city in China.

## Materials and methods

### Subjects

Data on incidence of fatal ICH from 1 June 2012 to 31 May 2014 were extracted from the acute stroke mortality database, which covers over 14 million permanent residents and was setup by Shanghai Municipal Center for Disease Control and Prevention (SCDC). In Shanghai, the onset date for all the stroke mortality cases was examined either by community doctors for deaths at home or by public health doctors for deaths in hospitals through checking hospital admission, discharge, and death records and interviewing family members. The demography information, stroke-related risk factors, and health conditions will be further collected and reported to SCDC if the patient died within 28 days after the onset date. All the reported information will be cross-checked with death certificates by staffs in SCDC.

### Definition of disease

In our study, we defined fatal ICH as those coded with I61 (ICD-10; WHO 1993) as underlying cause of death in the database. The subjects were stratified by sex, age (< 65, ≥ 65), and smoking status. For the smokers, we further classified them into two groups according to the median of amount of cigarette smoking per day. We calculated body mass index (BMI) as weight (in kilograms) divided by stature (in meters) squared (kg/m^2^). Subjects with BMI < 25 were classified as normal, and those with BMI ≥ 25 were classified as overweight. To examine the modifying effect of comorbid health conditions, hypertension (I10) and diabetes (E10–E14) were also defined.

### Source of air pollution and meteorological data

Daily concentration of PM_2.5_, sulfur dioxide (SO_2_), and nitrogen dioxide (NO_2_) was retrieved from the database of the Shanghai Environmental Monitoring Center, the government agency in charge of the collection of air pollution data in Shanghai. To allow adjustment for the effect of weather conditions, we also obtained daily mean temperature and humidity data from the Shanghai Meteorological Bureau.

### Statistical analysis

We conducted the time-stratified case-crossover approach to investigate the association between air pollution and fatal ICH. In our analysis, we selected control days matched on the same day of the week in the same month of the same year when a fatal ICH occurred. We performed conditional logistic regression, stratifying on each day, to obtain estimates of odds ratios (ORs) and 95% confidence intervals (CIs) associated with an interquartile range increase in mean daily air pollutant levels. We adjusted for meteorological conditions by fitting daily mean temperature and relative humidity by restricted cubic splines for the same lag as the pollutant in the model. As a sensitivity analysis, we examined the effect of air pollutants with different lag structures (from lag0 to lag3). Lag0 was defined as the current-day pollutant concentration, and lag1 refers to the previous day concentration, and so on. Lag03 refers to the average of lagged 0, 1, 2, and 3 days. All the reported effect estimates for PM_2.5_ in our study were adjusted by SO_2_ and NO_2_. We also calculated the 95% CI to test the statistically important significance of differences between effect estimates of the strata divided by potential effect modifiers, as elaborated by Zeka A in their work [20]. All the reported *P* values in our research are based on two-sided tests at *α* = 0.05 level. The analysis was performed using R, version 3.4.3 (R Development Core Team 2010).

## Results

### Subjects characteristics

There were a total of 5286 fatal ICH cases occurring in Shanghai between 1 June 2012 and 31 May 2014. Table 1 presents the summary statistics of daily fatal ICH. Among the 5286 subjects, 3833 (72.51%) were above 65, 3064 (57.96%) were male, and 1337 (25.29%) were with BMI ≥ 25. There were 1567 (29.64%) smokers in our study, and among them, 673 (42.95%) subjects smoked more than 10 cigarettes per day. 71.35% of the subjects were diagnosed with hypertension, and 17.51% were diagnosed with diabetes before fatal ICH onset. The average daily concentration of PM_2.5_ was 77.45 μg/m^3^ with an interquartile range of 58 μg/m^3^ during our study period (Table 2) (Additional file 1).

**Table 1:** Summary statistics of fatal ICH events by age, gender, BMI, smoking status, amount of cigarette smoking, and comorbid conditions in Shanghai, China, from 1 June 2012 to 31 May 2014

Variables	*N*	Percent (%)	Mean (SD)	Min	Max
Total	5286	100.00	7.24 (3.04)	0	17
Age < 65	1453	27.49	1.99 (1.43)	0	8
Age ≥ 65	3833	72.51	5.25 (2.51)	0	14
Male	3064	57.96	4.20 (2.23)	0	12
Female	2222	42.04	3.04 (1.79)	0	10
BMI < 25	3889	74.42	5.33 (2.64)	0	16
BMI ≥ 25	1337	25.58	1.83 (1.41)	0	8
Smokers	1567	29.78	2.15 (1.55)	0	10
Non-smokers	3695	70.22	5.06 (2.45)	0	14
Amount of smoking					
≤ 10 per day	876	56.55	1.20 (1.11)	0	7
> 10 per day	673	43.45	0.92 (0.98)	0	6
Hypertension					
With	3760	71.35	5.15 (2.41)	0	13
Without	1510	28.65	2.07 (1.53)	0	8
Diabetes					
With	921	17.51	1.26 (1.18)	0	6
Without	4338	82.49	5.94 (2.74)	0	16

**Table 2:** Air pollutant concentrations and weather conditions in Shanghai, China, from 1 June 2012 to 31 May 2014

Variables	Mean (SD)	Q1	Med	Q3	IQR
Meteorology					
Temperature (°C)	17.83 (9.37)	10.00	18.00	26.00	16.00
Humidity (%)	68.28 (12.91)	60.00	69.00	78.00	18.00
Pollutants (μg/m^3^)					
PM_2.5_	77.45 (52.48)	40.00	65.00	98.00	58.00
SO_2_	21.22 (13.56)	12.00	16.00	25.00	13.00
NO_2_	57.59 (24.28)	40.00	54.00	73.00	33.00

### Association between PM_2.5_ air pollution and fatal ICH incidence

Positive and statistically significant associations were found between PM_2.5_ and fatal ICH incidence (Table 3). Following the increase in lag2, we found an increase of 8% (2–15%) for fatal ICH incidence. After stratified by age, we found that the increase of PM_2.5_ was only associated with subjects above 65, with an 8% (1–16%) increase in incidence in lag2. When the subjects were stratified by sex or whether BMI ≥ 25, we found that increase of PM_2.5_ concentration in lag2 was both associated with a 10% (1–19%) increase of incidence in male and 20% (5–36%) in the BMI ≥ 25 group. However, no statistically significant differences were found between effect estimates of the strata divided by the above effect modifiers.

**Table 3:** Odds ratios of fatal ICH associated with an interquartile range increase in PM_2.5_ levels (OR and 95% CI)

Lag	Total	Age		Gender		BMI	
		< 65	≥ 65	Female	Male	< 25	≥ 25
0	1.00 (0.93, 1.08)	0.99 (0.86, 1.13)	1.01 (0.93, 1.1)	0.99 (0.89, 1.11)	1.01 (0.92, 1.11)	0.98 (0.90, 1.06)	1.10 (0.95, 1.26)
1	1.02 (0.96, 1.09)	1.05 (0.93, 1.19)	1.01 (0.94, 1.09)	1.05 (0.95, 1.16)	1.00 (0.92, 1.09)	1.01 (0.94, 1.09)	1.09 (0.96, 1.24)
2	1.08 (1.02, 1.15)*	1.08 (0.96, 1.22)	1.08 (1.01, 1.16)*	1.06 (0.97, 1.17)	1.10 (1.01, 1.19)*	1.05 (0.98, 1.13)	1.20 (1.05, 1.36)*
3	1.01 (0.95, 1.07)	0.96 (0.85, 1.08)	1.02 (0.95, 1.1)	1.01 (0.92, 1.12)	1.00 (0.92, 1.08)	1.00 (0.93, 1.07)	1.00 (0.88, 1.13)
03	1.06 (0.97, 1.15)	1.03 (0.87, 1.21)	1.07 (0.97, 1.18)	1.07 (0.94, 1.22)	1.05 (0.94, 1.17)	1.05 (0.95, 1.16)	1.10 (0.93, 1.31)

*P < 0.05

### Role of cigarette smoking and comorbid disease in effect modification

**Fig. 1:**
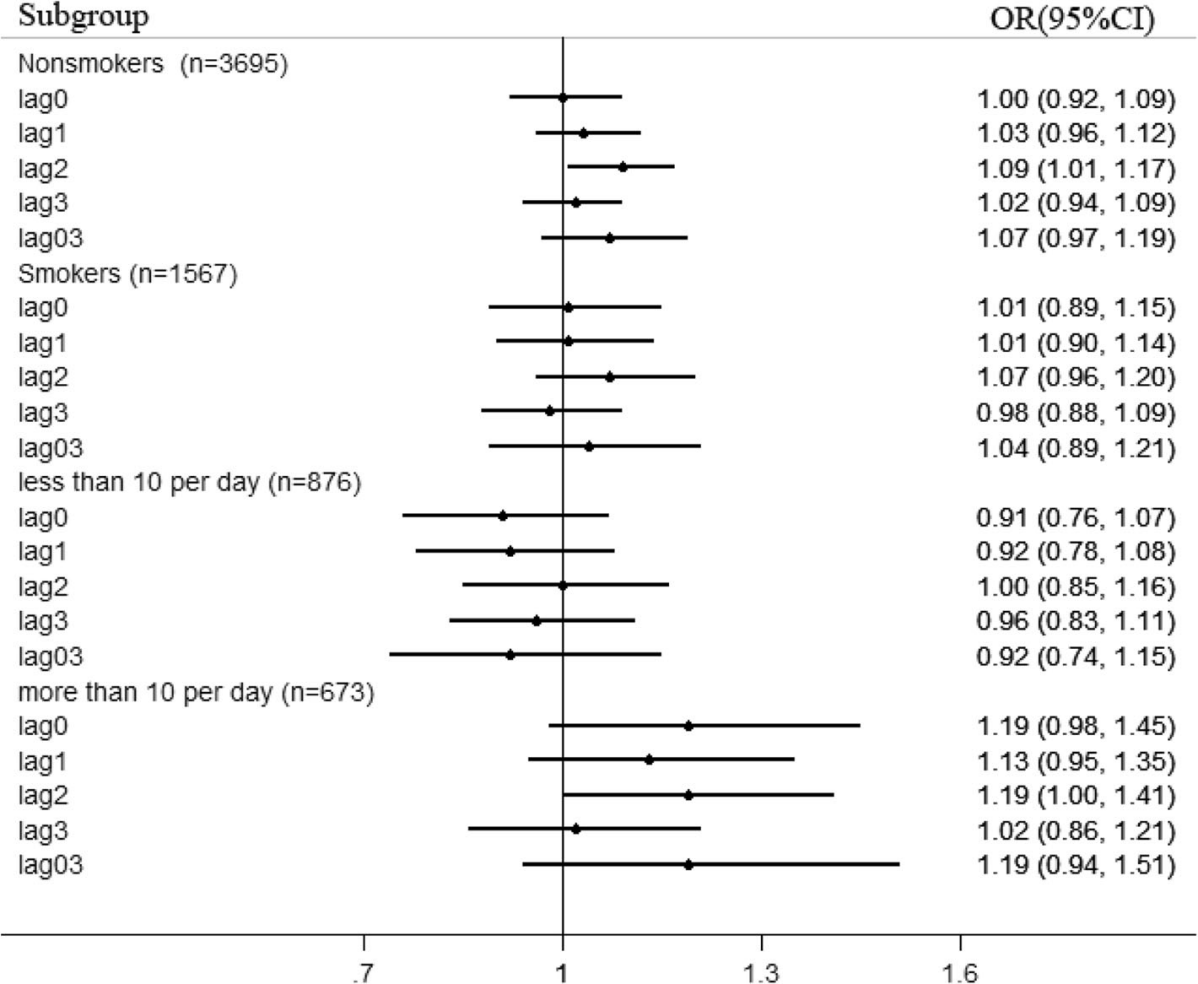
Effect modification by smoking status and amount of cigarette smoking

**Fig. 2:**
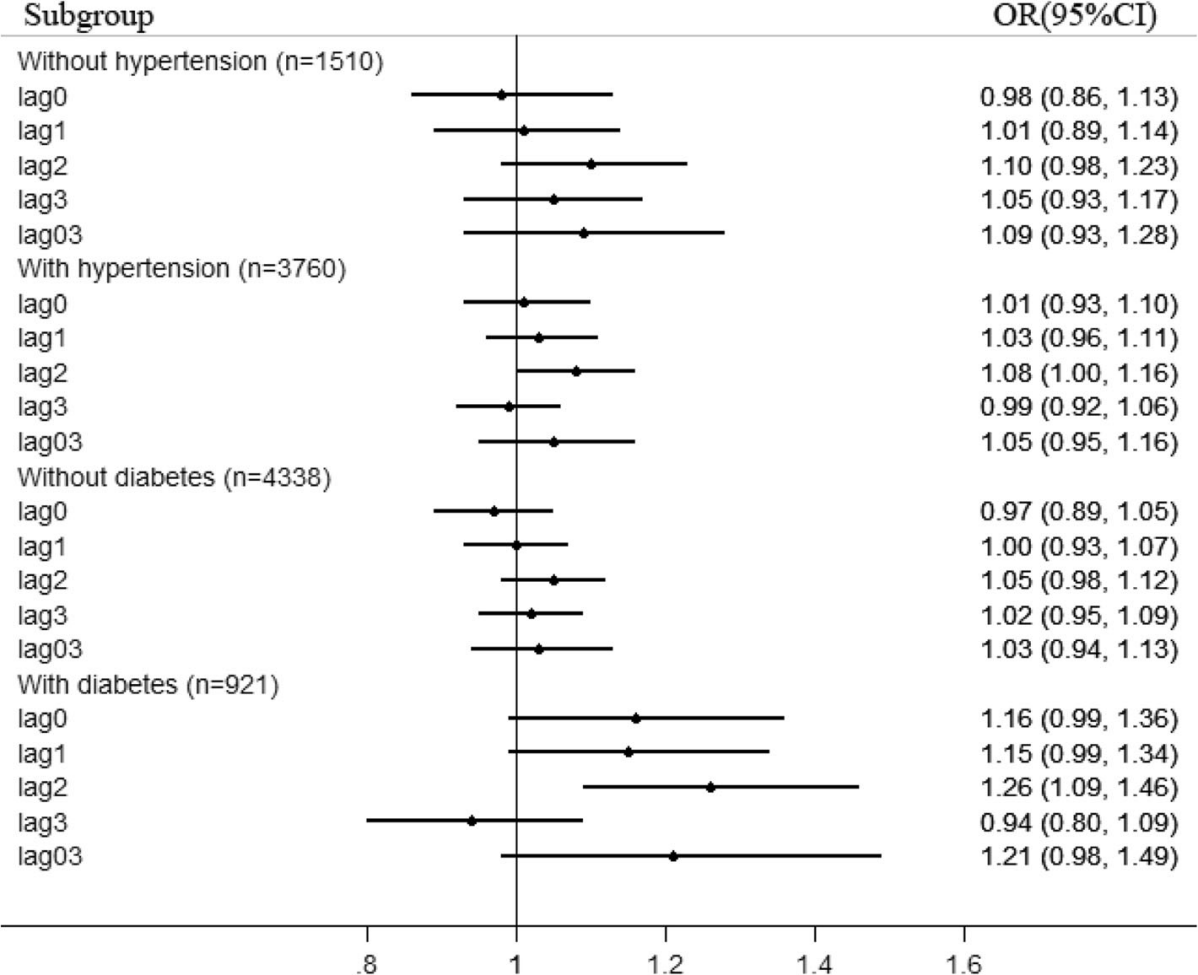
Effect modification by hypertension and diabetes

We classified the subjects by smoking status to further explore the possible susceptible population of ICH under PM_2.5_ exposure. As shown in Fig. 1, we found no statistically significant associations in smokers. Following the increase of PM_2.5_ in lag2, we observed a 9% (1–17%) increase of incidence in non-smokers. After classifying smokers into two groups according to the median of amount of cigarette smoking per day, we found that PM_2.5_ exposure significantly increased the risks of fatal ICH for subjects with more than 10 cigarette consumption per day, with an increase of 19% (0–41%) in incidence. As shown in Fig. 2, we also classified the subjects by the comorbid disease of hypertension. We observed a 8% (0–16%) increase of incidence in subjects with hypertension. It should be emphasized that the difference of effect estimates for strata divided by the above effect modifiers was insignificant. In contrast, following the increase of PM_2.5_ in lag2, an increase of 26% (9–46%) in incidence was observed for subjects with diabetes, which was markedly stronger than that among subjects without diabetes.

## Discussion

There has been limited information on association between PM_2.5_ exposure and ICH incidence. In a time-stratified case-crossover design, our study demonstrated that a transient increase in PM_2.5_ concentration was significantly associated with incidence of fatal ICH. Furthermore, findings from our study indicated that the presence of diabetes significantly modifies the effect of PM_2.5_ in triggering fatal ICH.

Several studies have examined associations between air pollutants and hemorrhagic stroke. Studies undertaken in America, Britain, and Canada found no significant associations [4, 21, 22]. Significantly positive associations were mostly found from China and Japan [10–13]. In contrast, studies concerning the relationship between air pollution and ICH were rarely conducted. In China, stroke mortality accounts for 19% of total mortality, compared with 8% caused by ischemic heart disease [9]. The relatively high stroke death rate offers us not only the possibility to explore the association between air pollution and the subtype of hemorrhagic stroke, but also enough power to examine the outcome.

In our study, we observed significant associations between PM_2.5_ and ICH incidence at lag2. Experimental studies in humans and animals have shown that exposure to PM_2.5_ can induce alterations in hemostatic factors, systemic inflammation, endothelial cell injury, and vascular dysfunction. These physiologic intermediates have typically been investigated in association with PM_2.5_ exposures within this time frame [23, 24].

Identification of sets of individuals who have an enhanced response to air pollutants may suggest possible mechanisms of physiological assault, as well as provide data that can be used for more detailed risk assessment [25]. Consistent with the previous study, the results of our study indicated that overweight and older than 65 may increase the risk of morbidity and mortality associated with ambient particulate matter [5, 26–29]. It has been speculated that blood pressure of patients with hypertension may be further increased by PM_2.5_ and lead to rupture of brain vessels [30]. Coughing was also suspected to be able to result in raised intracranial pressure (Valsalva effect) and rupture vulnerable aneurysms [10]. As a result, in our study, we also examined the possible modifying effect of hypertension and cigarette smoking. We found that the risk of ICH was significantly increased in subjects with hypertension, non-smokers, and those who smoked more than 10 cigarettes per day. However, no significant results were observed when we test the statistical significance of differences between effect estimates of the strata divided by the above potential effect modifiers. This means that the difference in effect estimates between age group, sex, hypertension, and cigarette smoking may be observed just by chance. In contrast, we found that the effect estimates observed in subjects with diabetes were markedly stronger than those in subjects without. Diabetes and airborne particles have been associated with increased thrombotic risk factors and increases in systemic markers of inflammation [31]. The possibly synergistic effect between them may explain the enhanced susceptibility of ICH found in our study and deserves to be further validated in other studies.

The registration of fatal stroke mortality in Shanghai as well as the collection of information at the individual level offered us major advantages over previous studies examining the effects of air pollutants on ICH incidence or mortality. There were also some limitations of our study. Firstly, the concentration of PM_2.5_, SO_2_, and NO_2_ in our study is from fixed outdoor air monitoring sites. This may cause exposure measurement error when used to represent individual exposure, leading to underestimation of the pollutant effects. Secondly, the demography information, health conditions, and onset date of the subjects were collected retrospectively, which may bring bias to our study. Finally, in view of most subjects in our study were old and may spend most of their time indoors because of their health conditions, there is the possibility that they will suffer from higher risks of ICH incidence when they were sufficiently exposed to air pollution.

## Conclusions

In summary, findings from the present study suggested that PM_2.5_ exposure was significantly associated with fatal ICH incidence. Patients with diabetes constitute the susceptible population of PM_2.5_-related ICH. Further toxicological studies are needed to elucidate the biological mechanisms and to confirm or refute our findings.

## Additional file

Additional file 1: **Table S1.** Correlations among PM_2.5_, SO_2_, and NO_2__. (DOCX 15 kb)_

## Abbreviations

BMI: Body mass index
HS: Hemorrhagic stroke
ICH: Intracerebral hemorrhage
IS: Ischemic stroke
NO_2_: Nitrogen dioxide
OR: Odds ratio
PM_2.5_: Particles ≤ 2.5 μm in aerodynamic diameter
SCDC: Shanghai Municipal Center for Disease Control and Prevention
SO_2_: Sulfur dioxide
