# Supplementary material for: Association between incidence of fatal intracerebral hemorrhagic stroke and fine particulate air pollution
Source: Environ Health Prev Med. 2019 Jun 1;24:38. doi: 10.1186/s12199-019-0793-9 (PMC6545210; doi:10.1186/s12199-019-0793-9)
Supplement: Supplementary file 1 — Table S1. Correlations among PM2.5, SO2, and NO2. (DOCX 15 kb) [file 12199_2019_793_MOESM1_ESM.docx]

Additional file 1: Table S1 Correlations among PM_2.5_, SO_2_ and NO_2_

|  | PM_2.5_ | SO_2_ | NO_2_ |
| --- | --- | --- | --- |
| PM_2.5_ | 1.00 | 0.76* | 0.77* |
| SO_2_ | 0.76* | 1.00 | 0.82* |
| NO_2_ | 0.77* | 0.82* | 1.00 |

*P<0.05
